# Medical linac photon skyshine: Monte Carlo calculations and a methodology for estimates

**DOI:** 10.1002/acm2.13543

**Published:** 2022-02-14

**Authors:** Patrick N. McDermott

**Affiliations:** ^1^ Beaumont Health System Royal Oak Michigan USA

**Keywords:** medical linear accelerators, Monte Carlo simulation, radiation shielding, roof shielding, skyshine

## Abstract

It has been shown that a widely quoted formula for estimating medical linac photon skyshine equivalent doses is erroneous. Monte Carlo calculations have been performed to develop an easy method for quickly and accurately estimating skyshine radiation levels and to gain improved physical insight into the skyshine phenomenon. Calculations of linac photon skyshine have been performed for 4, 6, 10, 15, and 18 MV beams for 10 × 10 cm^2^ and 40 × 40 cm^2^ fields and for a range of room dimensions and roof thicknesses. The effect of flattening filter free beams has been considered. Air kerma rates (AKRs) can be accurately fitted to a simple algebraic formula that is a function of the horizontal distance from the isocenter with a single energy dependent fitting parameter. The AKR, at a height of 1.3 m above level ground, reaches a local maximum at a distance *d*_max_ = 1.5*d_w_* + 1.1*h*, where *d_w_* is the horizontal distance from the isocenter to the outside of the side wall, and *h* is the vertical distance from the isocenter to the top of the roof. For thin roofs, low energy beams lead to significantly more skyshine than high energy beams because low energy photons are more easily scattered through large angles. In the absence of a roof, the maximum skyshine dose rate is on the order of 8 × 10^−7^ times the dose rate at isocenter. The average energy of the skyshine photons is about 0.15 MeV, and it is remarkably independent of almost all parameters. A simple methodology is outlined for the evaluation of photon skyshine.

## 1 INTRODUCTION

For linac radiotherapy facilities having minimal roof shielding, the possibility that appreciable photon and neutron radiation scattered by the air may reach ground level must be considered. This is referred to as skyshine. For outdoor areas, the occupancy factor will be low, perhaps *T* = 1/20 to 1/40 depending on activity in these areas. Skyshine may, however, also affect indoor areas, particularly adjacent rooms but also nearby single story buildings where the occupancy factor may be 1.0. The DIRAC database of the International Atomic Energy Agency (IAEA) reports that there are currently over 18 000 megavoltage radiation therapy units worldwide.^1^ Skyshine may be an important consideration for a large percentage of these units.

NCRP Report No. 151, IAEA Report No. 47 and IPEM Report 75 recommend that skyshine should be considered when roof shielding is minimal; the IAEA report however does not provide any methodology to accomplish this.^2^, ^3^, ^4^ The NCRP151 and IPEM75 reports provide a formula to estimate the photon radiation level associated with skyshine (Equation (5.1) in NCRP151), but it has been shown that this formula is grossly incorrect.^2^, ^3^, ^4^, ^5^ The formula is in error by as much as an order of magnitude.^5^, ^6^ This formula is not even qualitatively correct in that it fails to predict the observed local maximum in skyshine radiation level as a function of horizontal distance from the linac isocenter. The origin of this formula is somewhat murky. The original references are not easily accessible. NCRP151 quotes McGinley, and McGinley and Martin quote NCRP151.^7^ According to McGinley this formula is based on measurements made with a Cs‐137 and Co‐60 source placed in a hole in the ground.^7^ For a linac vault, the radiation source has a broad energy spectrum with a higher average energy than these isotopes. Furthermore, it is collimated, and the radiation issues from the rooftop, not from a hole in the ground.

**FIGURE 1:**
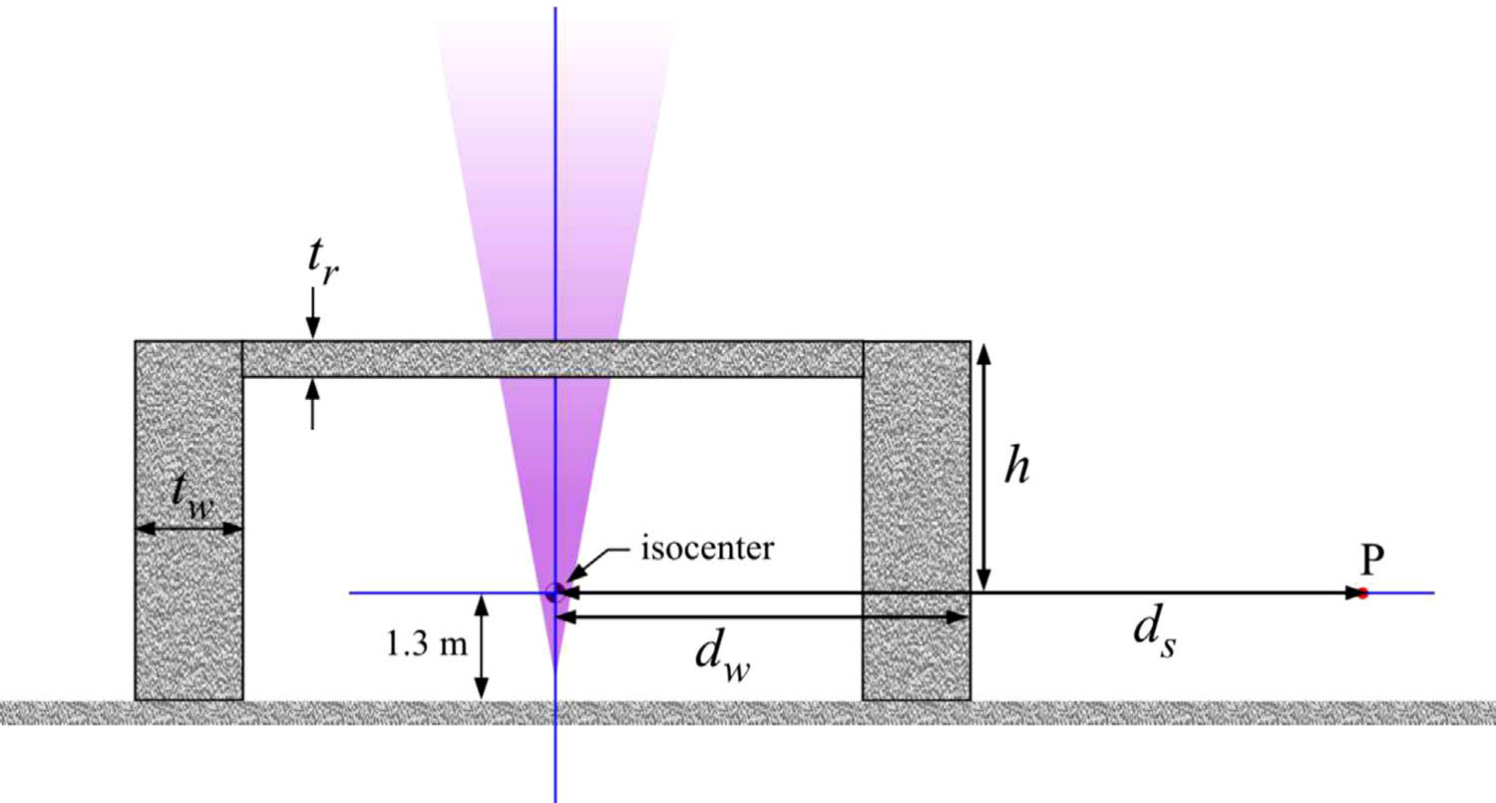
The geometry of the linac vault drawn to scale for the nominal room dimensions discussed in the text (*d_w_* = 5.0 m, *h* = 3.0 m). The observation point is labeled **P** and is at distance *d_s_* from isocenter

It has been shown that photon skyshine radiation levels, measured as a function of horizontal distance from the isocenter, can be accurately fit with a single energy dependent parameter using a simple algebraic formula derived by considering single photon scattering.^8^ The instantaneous air kerma rate (AKR) in units of nSv/s is given by:

(1) $$\begin{array}{cc} {\dot{K}}_{a}=\; & k\left(\frac{F_{0}}{400}\right)B_{\mathrm{xs}}\left(\frac{{\dot{D}}_{0}}{400}\right)\frac{1}{d_{s}}\\ & \times \left[2{\left(1+x^{2}\right)}^{3/2}-x\left(2x^{2}+3\right)\right], \end{array}$$

where *k* is the energy dependent fitting parameter, *F*_0_ is the field area at isocenter in units of cm^2^, *B_xs_* is the roof transmission factor, and ${\dot{D}}_{0}$ is the dose rate at isocenter expressed in cGy/min, *d_s_* is the horizontal distance from the isocenter, $x=h/(d_{s}-d_{w})$, *d_w_* is the horizontal distance from the isocenter to the outer surface of the side wall, *h* is the vertical distance from the isocenter to the top of the roof. The geometry is shown in Figure 1. Previously quoted values of the fitting parameter are somewhat uncertain because of uncertainties in the roof transmission and because of the limited measured data available.^8^ Monte Carlo (hereafter MC) calculations may provide more accurate values of this parameter across the full range in beam energy and eliminate the complication of uncertain roof transmission.

Measured AKRs show that the skyshine exhibits a local maximum (designated here as *d*_max_) as a function of horizontal distance from the isocenter.^6^, ^9^ Equation (1) can be analyzed to locate the predicted value of *d*_max_ at the height of the isocenter, assumed to be 1.3 m above level ground. This value is found to be approximately linear as follows (over the range 0.3 < *h/d_w_* <  1.2):

(2) $$d_{\max}\approx 1.5\;d_{w}+1.1\;h.$$

This formula predicts observed/measured values of *d*_max_ within an accuracy of better than 10%.^6^, ^9^

The purpose of this paper is to provide greater understanding of the photon skyshine phenomenon, in particular for medical linacs, and to provide the medical or health physicist with a relatively simple methodology to quickly, easily, and accurately estimate photon skyshine radiation levels using Equations (1) and (2).

MC calculations are reported here for beam energies of 4, 6, 10, 15, and 18 MV, for field sizes of 10 × 10 cm^2^ and 40 × 40 cm^2^ and for several sets of room dimensions. Neutron skyshine may also be important at high energies but is not addressed here. We have evaluated the effects of the use of flattening filter free (FFF) beams and have also considered the effects of air density (and hence altitude).

There have been numerous publications on MC calculations of photon skyshine. The majority of this literature relates to high energy accelerators or the storage of radioactive material and is therefore of little relevance to medical linac facilities. The photon energies studied are not pertinent to medical linacs, and the geometries and distances considered are quite different. Kong et al. report on MC calculations of linac photon skyshine produced by 9, 15, and 21 MV linac beams.^10^ The smallest distance from the isocenter that they refer to is 20 m. This is generally well beyond the distance at which the skyshine reaches a maximum. They define Ω as “the solid angle between the source and the vertical wall.” This appears to be a misunderstanding of this quantity as defined in NCRP151.^2^ There is no mention of the beam field size. The cutoff energy was set to 100 keV. This value is too high considering that the average energy of the ground level skyshine photons reported here is about 150 keV.

## 2 MATERIALS AND METHODS

The MC code (hereafter referred to as MCLS) was written in the Mathematica (v 8.0.1.0) programming language specifically for the purpose of evaluating skyshine and side scatter. The MCLS code does not include charged particle transport. The linac vault is assumed to be cylindrically symmetric with an outer wall diameter *d_w_* and a distance from isocenter to rooftop of *h* (see Figure 1). The beam central axis is coaxial with the axis of the cylinder. The cylindrical symmetry is exploited by tallying AKR for thin rings in a horizontal plane (at a specified height above ground level) that are coaxial with the cylindrical vault. The condition of cylindrical symmetry precludes examination of the skyshine when the beam central axis is obliquely incident on the roof. The advantage of writing a dedicated code is complete and total control over all aspects. The disadvantage is that the validity of the code is unproven. For this reason, extensive validation tests have been conducted, some of which are discussed below.

The following are some additional assumptions that are inherent in the MCLS code.

1. The geometry is cylindrically (or axially) symmetric (see Figure 1). The results presented here therefore do not apply if the beam is obliquely incident on the roof or if the field cross section is significantly lacking in symmetry about the central axis.
2. The linac beam is conical (circular in cross section, consistent with 1, above), not pyramidal as for a square field. The field size reported here is the equivalent square field size, that is, a 40 × 40 cm^2^ field is circular in cross section and has an area of 1600 cm^2^.
3. The emitted photon fluence is isotropic over the cone, and the beam spectrum does not vary off‐axis.
4. Photons having energy less than 10 keV are assumed absorbed on the spot.
5. The density of air does not vary with height (see below).
6. Pair annihilation photons are ignored. Photons are “killed” if they undergo pair production. The highest beam energy considered is 18 MV. The average energy of a photon in an 18 MV beam is approximately 6 MeV. The ratio of the linear attenuation coefficient for pair production alone to the total linear attenuation coefficient is 0.1 for 6 MeV photons in air.
7. The linac side walls and the roof are composed of ordinary composition concrete with a density of 2.3 g/cm^3^.
8. The ground is composed of concrete.

No variance reduction techniques have been used. Mass attenuation coefficients were obtained from the NIST XCOM database.

Due to cylindrical symmetry, all dosimetric quantities are independent of the azimuthal angle and are tallied in flat coaxial rings (1.3 m above ground level) with constant width $\Delta r=r_{n+1}-r_{n}$. The incremental contribution to the air kerma for a photon crossing a ring is:

(3) $$\Delta K_{a}=E_{\gamma }\Delta \Phi \frac{\mu_{en}}{\rho }$$

where ΔΦ is the fluence due to a single photon crossing. The contribution to the fluence from a single photon crossing a ring is 1/(projected area of the ring). The projected area of the ring is the area of the ring projected onto a plane perpendicular to the direction of motion of the photon. The unit normal vector to the ring is $\hat{k}$, and the direction of motion is given by the vector consisting of the direction cosines (*u*, *v*, *w*). The contribution to the fluence from a crossing photon is:

(4) $$\Delta \Phi =\frac{1}{2\pi r_{n}\left[1+\frac{\Delta r}{2r_{n}}\right]\left|w\right|\;\Delta r}.$$

The same expression can be derived from the Chilton definition of the fluence (Σdl/V), where *dl* is the track length of the photon through a thin ring of thickness Δ*z*, and *V* is the volume of the ring.

The assumed composition of the roof, side walls, and ground is not expected to be a significant factor provided that *B*_xs_ is the same.

The importance of atmospheric density stratification can be assessed by comparing the density scale height with the mean free path of the photons. The density scale height at sea level is given by ${\left(\frac{1}{\rho }\frac{d\rho }{dz}\right)}^{-1}$ = 6.6 km. The average energy of the photons in an 18 MV beam is roughly 6 MeV. The mean free path, 1/*μ*, for 6 MeV photons in air at sea level is 0.33 km. The ratio of these two length scales is 0.05, and therefore the effect of atmospheric density stratification is expected to be small. The effect of the value of the absolute air density is discussed later.

### 2.1 MCLS code validation

It is difficult to find a simple, straightforward test of an MC skyshine code. Comparison with linac measurements is complicated by the fact that studies reporting such measurements do not provide sufficient geometry and composition detail necessary for MC calculations.

**FIGURE 2:**
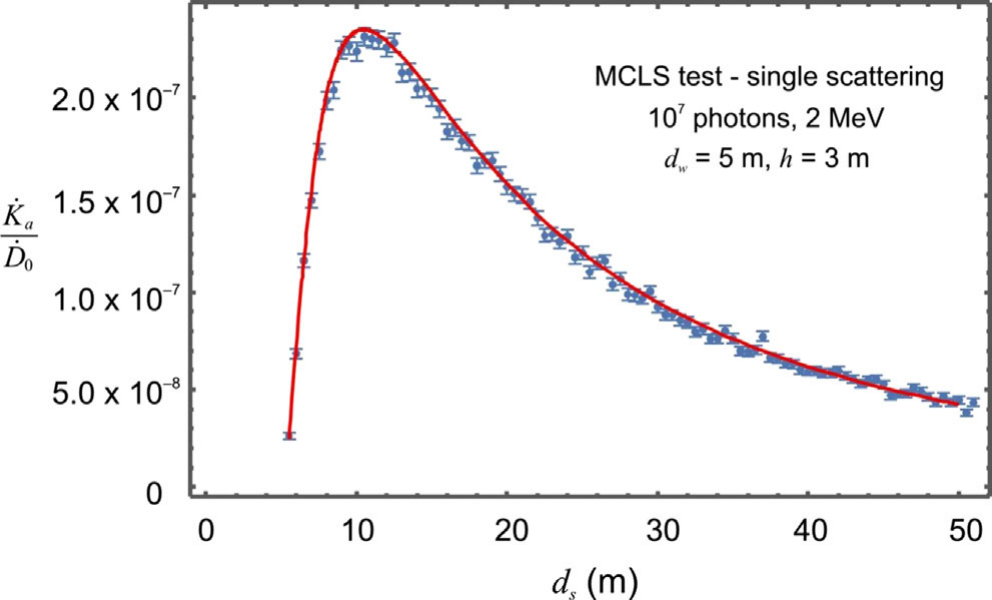
A test of the MCLS code. This graph shows the scaled air kerma rate as a function of horizontal distance for singly scattered 2 MeV photons that have been projected upward along a vertical axis. The MCLS results (points with error bars) are for 10^7^ histories. The dimensions of the linac vault are *d_w_* = 5 m and *h* = 3 m. The solid (red, on‐line version only) curve results from the evaluation of an integral formula for monoenergetic singly scattered photons. This integral has been evaluated numerically for the same parameters as the MCLS code

The MCLS code was first tested against a semi‐analytic model for monoenergetic photons projected up along the vertical central axis in the absence of a roof and with restriction to single scattering. Under these circumstances, the air kerma as a function of horizontal distance from the isocenter (at the same height as the isocenter) can be expressed as an integral.* This integral can be evaluated numerically for comparison with the output of the MCLS code for singly scattered photons. This comparison is shown in Figure 2 for 10^7^ histories, 2 MeV photons, and vault dimensions of *d_w_* = 5 m and *h* = 3 m. The 2 MeV photon energy is approximately the average energy of the photons in a 6 MV linac beam. The AKR,${\dot{K}}_{a}$, has been scaled by dividing by the dose rate at isocenter for a 10 × 10 cm^2^ field with 10^7^ photons (${\dot{D}}_{0}$). The MCLS air kerma is in excellent agreement with the numerical integration as shown in Figure 2.

A definitive test of the MCLS code is based on a benchmark skyshine experiment conducted at Kansas State University in the late 1970s.^11^ A concrete silo was constructed in the form of a hollow cylinder with an outer radius of 2.16 m and a height of 2.3 m above ground level. The thickness of the concrete was 0.91 m. Cobalt‐60 sources were placed inside the silo on the symmetry axis of the cylinder (at a height of 2.0 m). Measurements were made with the silo open (no roof) and with a concrete roof of thickness 43 cm.

Skyshine exposure rate measurements were made outside the silo at various radial distances from the central axis of the cylinder at a height of 1.0 m above the ground. In a paper published in 1993, these measurements were compared with MC calculations for an open roof with the well‐known MCNP code.^12^, ^13^ The MCNP calculated exposure rates were found to be in good agreement with the KSU measured values. At a distance of 50 m, the agreement was within 1.2%. This was well within the statistical uncertainty of the MCNP calculations.

The radial distances for the MCNP calculations ranged from 10 to 700 m. It is not clear why MCNP calculations were not made for distances less than 10 m, especially in view of the fact that the maximum exposure rate is expected to be found at a distance of less than 10 m. Equation (2), although not strictly applicable in this case, predicts *d*_max_ ∼ 5 m. The authors considered both MCNP calculations that include “in silo” scattering and some that exclude this. In silo scattering accounts for photons emitted by the Co‐60 source that are scattered inside the silo (from the concrete walls and support structure). The structure supporting the source is somewhat complex in geometry and composition, involving a cask and an underlying steel plate.

The MCLS code has been used to simulate this same geometry and composition. It is assumed that the photons are emitted isotropically with discrete energies of 1.17 and 1.33 MeV. The bin size for the skyshine tally is 0.5 m in radial extent, which is the same as used by MCNP. For this bin size, each bin captures a fraction of about 10^−3^ of the number of emitted photons. Ground reflection contributes about 1/3 of the exposure measured at a height of 1.0 m above ground level.

The MCNP paper makes the point that even a “simple” benchmark experiment is not so simple, as the calculated exposure rates depend on the details of the in silo scatter and to a lesser extent even the degradation of the source photon spectrum by the source container placed inside the silo.^13^ The authors state that the in silo scattering accounts for a surprising 16% of the exposure at a distance of 50 m in the absence of a roof. Seventy‐five percent of the in silo scatter is due to the source cask and the steel plate supporting the cask. We are unable to duplicate all the detail of the interior of the silo. We have assumed that it is an empty concrete cylinder, although scattering off the walls is included.

The MCNP paper quotes an exposure rate (with the roof open) of (20.1 ± 0.14) μR h^−1^ Ci^−1^ at a radial distance of 50 m in the absence of any in silo scatter.^13^ This is the only radial distance for which results are quoted in the absence of in silo scatter. The exposure rate calculated by MCLS for 10^7^ emitted photons (air density set to 1.12 × 10^−3^ g/cm^3^) under these circumstances is (19.9 ± 0.45) μR h^−1^ Ci^−1^. It thus appears as if the MCLS results are in agreement with MCNP within statistical uncertainty.

**FIGURE 3:**
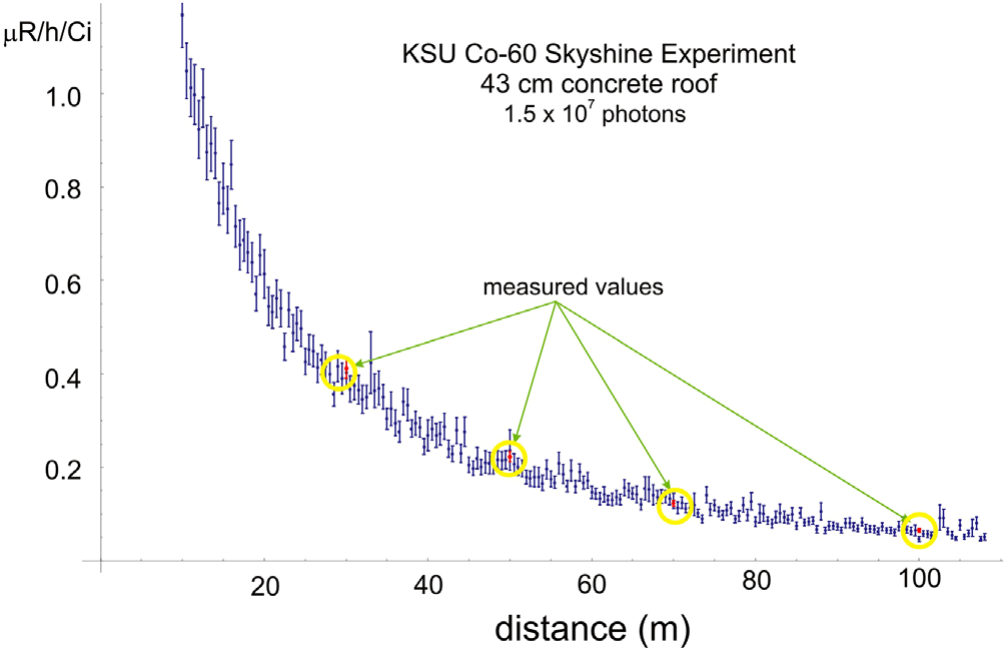
MCLS validation. The exposure rate per Ci as a function of distance from the central axis of the cylindrical silo for the Kansas State University (KSU) skyshine experiment in which there is a 43‐cm concrete roof. The MCLS calculated values are represented by the blue (on‐line version only) error bars. The KSU measured values are circled so that they may be seen among the blizzard of error bars

In the most definitive test of the code, MCLS has also been compared to measured exposure rates with a 43‐cm thick concrete roof lid.^11^ The results are shown in Figure 3. The air density is set to 1.21 × 10^−3^ g cm^−3^ (the density reported at the time of the measurements), and the number of photon histories is 1.5 × 10^7^. Figure 3 shows that the MCLS exposure rates agree with the measurements within the error bars.

**FIGURE 4:**
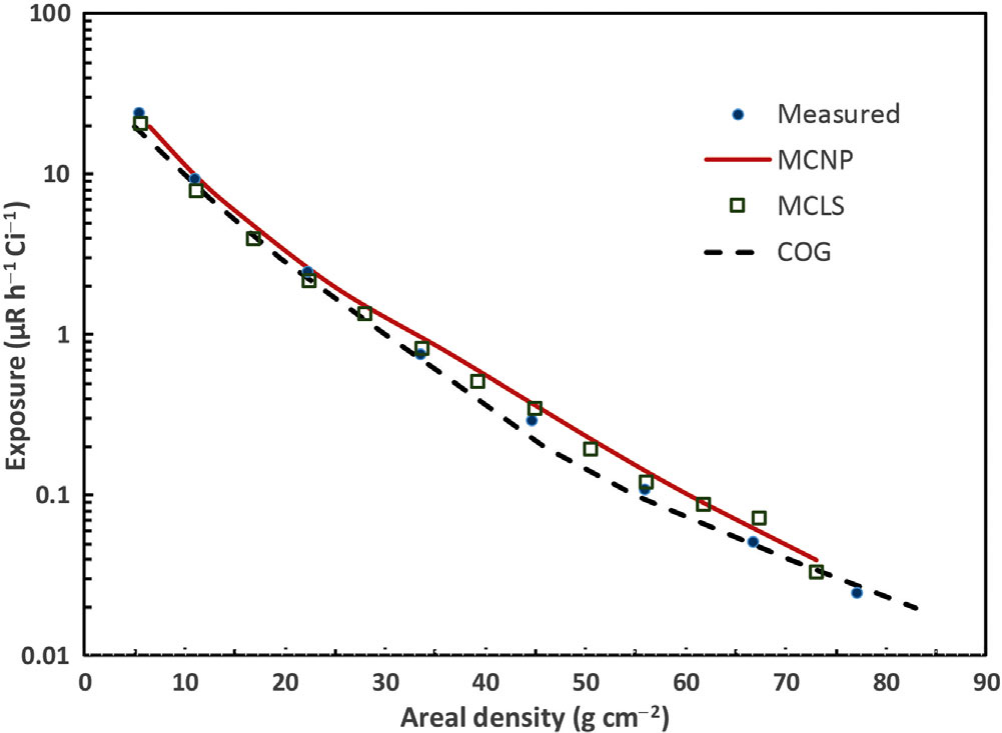
MCLS validation by comparison with the Los Alamos benchmark study of MCNP. The Los Alamos study compared MCNP and “COG” calculations against the Kansas State University skyshine measurements in the absence of a roof. The “COG” data are the result of Boltzmann transport calculations. The exposure rate per Ci is plotted as a function of the areal density of the air from the central axis of the cylinder. The MCLS calculated values lie mostly between those from the MCNP code and COG

Further validation of the MCLS code is provided by a comparison with the Los Alamos MCNP benchmark study.^12^ As part of the benchmark validation of the MCNP code, the Los Alamos authors compared MCNP and COG calculations of skyshine radiation levels for the KSU experiment (with no concrete roof) with measured values. The COG results are based on a solution of the Boltzmann transport equation. Figure 4 shows a plot of the exposure rate (per Ci) as a function of the areal density (units g cm^−2^) measured from the central axis of the cylinder at a height of 1 m above ground level. The areal density is the radial distance from the central axis of the cylinder multiplied by the density of the air. The figure shows that there is good agreement between the MCNP, MCLS, and COG calculations and the measurements. The MCLS values tend to lie between the MCNP and COG values. The accuracy of the calculated values can be assessed by computing the average value of |c−m|/m, where *c* is the calculated value, and *m* is the measured value. The values of this quantity are 15%, 13%, and 22% for MCLS, COG, and MCNP, respectively. MCLS agrees better with measurements than MCNP in this case.

### 2.2 Incident energy spectrum

The linac incident beam differential fluence spectra are taken from Sheikh‐Bagheri and Rogers, for 4, 6, 10, 15, and 18 MV Varian linacs.^14^ Skyshine and side scatter radiation levels are not especially sensitive to beam energy, and therefore the results reported here are expected to be applicable to Elekta as well as Varian linacs. An example of an MCLS sampled beam spectrum is shown in the Results section.

### 2.3 Vault dimensions

The choice of room dimensions can be assessed by consulting linac manufacturer's specifications. The Varian “Designers Desk Reference” quotes dimensions for a typical room configuration.† The Elekta Oncology Products “Site Planning Guide” quotes minimum room dimensions.‡ These dimensions are summarized in Table 1. The dimensions are similar for Varian and Elekta.

**TABLE 1:** Linac vault inner dimensions

Linac	Isocenter to rear wall (m)	Isocenter to side wall (m)	Floor to inner ceiling height (m)
Elekta	3.3	3.0	3.2
Varian	3.4	3.1	3.4

All barriers are assumed to be concrete. For an 18 MV treatment room, the side walls will have a thickness of about 1.8 m to shield against the primary beam. This leads to *d_w_* = 3.1 m + 1.8 m = 4.9 m for this barrier. The rear wall is exposed only to leakage and patient scatter and is assumed to be 1.0 m thick. This implies *d_w_* = 4.1 m for the rear wall. A standard nominal value of *d_w_* = 5.0 m for the side wall shall be assumed. For a ceiling thickness of 0.7 m and an isocenter height of 1.3 m from the floor, the distance from isocenter to the top of the roof is *h* = 2.8 m. A standard nominal value of *h* = 3.0 m has been chosen. The side walls are assumed to be 1.5 m thick throughout. In addition to calculations for (*d*_w_, *h*) = (5 m, 3 m), we have also done calculations for (*d*_w_, *h*) = (5 m, 5 m) and (10 m, 3 m).

We now consider values to adopt for the roof thickness *t_r_*. The extreme limits are zero thickness on the one hand, and on the other limit, whatever thickness is necessary to meet regulatory or ALARA guidelines for occupancy. At the high end, the thickness may be estimated as follows. Assume the NCRP151 recommended permissible equivalent dose for an uncontrolled area of *P* = 20 μSv/wk, and *W* = 500 Sv/wk, *U* = 0.25, *T* = 1/40 (rooftop), 18 MV, *d* = 4.3 m (target to point of interest distance).^1^ Under these assumptions the necessary concrete thickness is about 180 cm. The *B*_xs_ needed to achieve this is about 5.0 × 10^−5^. Skyshine will become relevant only when the thickness is considerably less than this value. We have chosen thicknesses of 0, 46 cm (1.5 ft) and 61 cm (2.0 ft) for 4 and 6 MV and 0 cm, 61 cm and 92 cm (3.0 ft) for energies above 6 MV.

## 3 RESULTS

**FIGURE 5:**
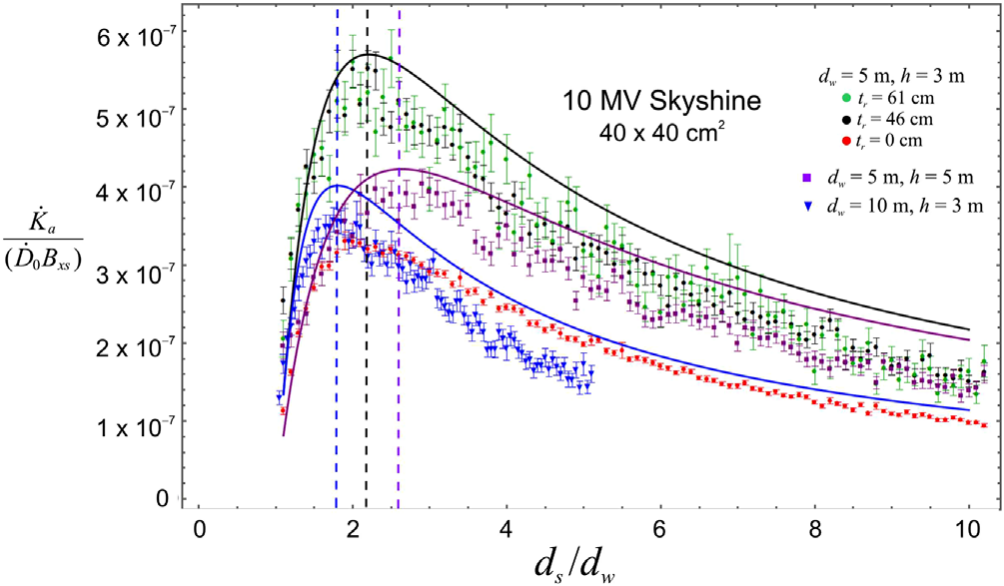
The scaled air kerma rate as a function of the dimensionless horizontal distance (*d_s_*/*d_w_*) from the isocenter for 10 MV photons for room dimensions (in meters) of (*d_w_*, *h*) = (5, 3), (5, 5), (10, 3) and for roof thicknesses (concrete) of 0 cm, 46 cm, and 61 cm. The solid curves show the conservative fits to Equation (1) for *k* = 100 and for the three combinations of (*d_w_*, *h*). The dashed vertical lines indicate the locations predicted by Equation (2) at which the air kerma rate is a maximum. The number of photon histories is 10^7^

We have computed linac skyshine relative AKRs (${\dot{K}}_{a}/\left({\dot{D}}_{0}B_{xs}\right)$ = AKR divided by the dose rate at isocenter for a 10 × 10 cm^2^ field size and by the roof transmission), for energies of 4 MV, 6 MV, 10 MV, 15 MV, and 18 MV. Values of the fitting parameter *k* in Equation (1) have been estimated based on these calculations for a variety of roof thicknesses and for various values of *d_w_* and *h*. The values of *k* are compiled in Table 2. These values are very conservative. We have chosen fits that tend to skirt the top of the MCLS AKR error bars (as shown in Figure 5). The values of *k* tend to significantly overestimate the MCLS AKR at distances beyond about 15–20 m. When using the values of *k* in Equation (1), the values of *B*_xs_ must be computed from the values of TVL_1_ and TVL_e_ found in NCRP151. The reason for this is that the *k* values were computed this way, and therefore the only way to accurately reconstruct the MCLS AKR is to use the NCRP151 calculated *B_xs_*.

**TABLE 2:** Values of fitting parameter *k*

Energy (MV)	4	6	10	15	18
*k*	150	140	100	95	85

Most of the results presented here are based on 10^7^ photon histories. The bin size (in *d_s_*) used for tallying air kerma has been set to 0.5 m. An example of the statistical accuracy achieved is provided by the case: 10 MV, roof thickness of 61 cm, 40 × 40 cm^2^ field size, (*d_w_*, *h*) = (5 m, 3 m). In this case, the statistical accuracy is about 10% (one standard deviation). When there is no shielding in the roof, reasonable statistical accuracy can be obtained with as little as 10^6^ histories.

The values of *k* in Table 2 are smaller than the values quoted in a previous paper.^8^ This is because, at least in part, those values were based on fits using measured values of *B*_xs_ for a 10 × 10 cm^2^ field. The roof transmission factors measured by Elder et al. and those calculated by MCLS show that *B*_xs_ is almost a factor of two larger for a 6 MV 40 × 40 cm^2^ field size.^9^

Figure 5 shows a graph of the scaled AKR, ${\dot{K}}_{a}/\left({\dot{D}}_{0}B_{xs}\right)$, as a function of *d_s_*/*d_w_* (at height of 1.3 m above ground level) for 10 MV photons, for concrete roof thicknesses of 0 cm, 46 cm, and 61 cm, for (*d_w_*, *h*) = (5 m, 3 m) and for a 40 × 40 cm^2^ field size. Also shown are results for (*d_w_*, *h*) = (10 m, 3 m) and (5 m, 5 m) for roof thickness 46 cm. In addition, the fits to Equation (1) with *k* = 100 are shown for the three different combinations of (*d_w,_ h*). The barrier transmission *B*_xs_ is computed using NCRP151 parameters for TVL_1_ and TVL_e_. The maximum AKR is on the order of 3.5 × 10^−7^ times the dose rate at isocenter (for *B*_xs_ = 1). The vertical lines in Figure 5 show the location of the maximum AKR predicted by Equation (2). The number of photon histories is 10^7^. The graphs for other beam energies have a similar appearance.

For 10 MV, Figure 5 shows that *k* = 100 provides a conservative fit (*not the best fit*) for roof thicknesses of 46 cm and 61 cm and different values of (*d_w_*, *h*). The values of *k* in Table 2 have been chosen so that the AKR calculated using Equation (1) will exceed the MCLS calculated values under almost all circumstances. The differences between AKR computed by Equation (1) and by MCLS are greatest at large distances. This may be due to the fact that Equation (1) does not account for attenuation of scattered photons. The location of the relative maximum is accurately predicted by Equation (2) as shown by the vertical lines in Figure 5.

The scaled AKR is considerably lower, by almost a factor of two, for an open roof (*B*_xs_ = 1, see the *t_r_*_ _= 0 data in Figure 5) than for a thickness of 46 cm or 61 cm, and therefore Equation (1) will overestimate the AKR by the same factor. Unless the roof is very thin, there is a substantial change in the phase space distribution of photons upon passing through the roof because the photons undergo multiple scattering events. For this reason, the values of *k* in Table 2 are not very accurate for thin roofs, especially for 10 MV and above, and will overestimate the AKR by almost a factor of 2.

We define a “thick” roof versus a “thin” roof. A thick roof is one for which $\bar{\mu }\;t_{r}>>1$ where $\bar{\mu }=\ln10/\mathrm{TV}L_{1}$, and TVL_1_ is the first tenth value layer. The quantity $\bar{\mu }\;$ is a sort of broad beam polyenergetic attenuation coefficient. For 4 MV (concrete), $1/\bar{\mu }$ is approximately 8 cm and for 18 MV it is 15 cm. The thicker the roof, the more important multiple scattering becomes.

When the roof is thin, the skyshine from low energy beams will be greater than from high energy beams. For example, when *B*_xs_ = 1, for a 40 × 40 cm^2^ field size, (*d_w_*, *h*) = (5 m, 3 m), at *d*_max_ the 18 MV skyshine ${\left({\dot{K}}_{a}/{\dot{D}}_{0}\right)}_{\max}$ = 2.1 × 10^−7^ whereas for 4 MV ${\left({\dot{K}}_{a}/{\dot{D}}_{0}\right)}_{\max}$ = 7.5 × 10^−7^, almost a factor of four larger. This is due to the fact that low energy photons are more easily scattered through large angles than high energy photons. As the roof becomes thicker, this relationship will shift so that higher energy beams contribute more because the amount of radiation emerging from the roof will decline much faster for low energy photons. For roof thickness, *t_r_* (concrete or equivalent), of less than roughly 30 cm, 4 MV and 6 MV skyshine radiation AKR are about the same, and they dominate other energies. The maximum AKR is relatively independent of energy for 30 cm ≲ *t_r_* ≲ 50 cm. For *t_r_* ≳ 50 cm,the highest beam energy available will dominate.

**FIGURE 6:**
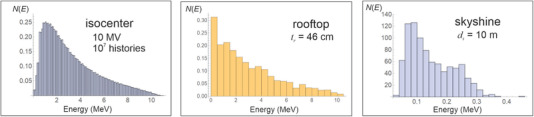
The energy spectrum for an MCLS run with 10^7^ histories for the 10 MV beam (40 × 40 cm^2^) at three locations: isocenter, roof top, and near ground level (height 1.3 m, horizontal distance 10 m), for (*d_w_*, *h*, *t_r_*) = (5 m, 3 m, 46 cm). The mean energies are 3.2 MeV at isocenter, 2.9 MeV at the rooftop, and 0.15 MeV near ground level

The energy of skyshine photons is remarkably independent of the linac beam energy, field size, and roof thickness. This can be understood by considering the physics of Compton scattering. When the energy of a photon *E_γ_* >> *m*_0_ *c*,^2^ the energy of a scattered photon is independent of *E_γ_*. At a distance of *d_s_* = 10 m (height of 1.3 m above ground), the average energy of these photons for an open roof is about 0.18 MeV for (*d_w_*, *h*) = (5 m, 3 m) for all energies (4 MV to 18 MV) and field sizes. The energy declines slightly to about 0.15 MeV for a thick roof. Some of the beam spectra show a slight peak at about 0.25 MeV, which is the energy of scattered high energy photons that have scattered through 180^o^. This likely represents singly scattered photons. An example of the energy distribution of the photons for a 10 MV beam is shown in Figure 6 at the isocenter, at the rooftop after traversing 46 cm of concrete and near ground level at a distance of *d_s_* = 10 m.

A significant fraction of the skyshine at a height of 1.3 m above the ground is a result of ground reflection. This has been evaluated by running a version of the MCLS code in which any photon striking the ground is “killed.” This has been done for 10 MV, 40 × 40 cm^2^, (*d_w_*, *h*, *t_r_*) = (5 m, 3 m, 0 cm) and 10^7^ histories. The skyshine AKR at a distance of 10 m is ${\dot{K}}_{a}/{\dot{D}}_{0}$= (3.18 ± 0.05) × 10^−7^. In the absence of ground reflection at a distance of 10 m, ${\dot{K}}_{a}/{\dot{D}}_{0}$ = (2.71 ± 0.04) × 10^−7^. Under these circumstances,ground reflection represents about 15% of the total skyshine.

The ratio of the skyshine AKR from singly scattered photons to that for multiply scattered photons varies with distance *d_s_*. This ratio peaks with a value of roughly ½ at *d*_max_ and declines slowly for *d_s_* >* d*_max_. In the “shadow” of the linac side wall (*d_s_* <* d*_max_), it declines rapidly with decreasing *d_s_* as one might expect as it is much more difficult for singly scattered photons to reach this region. For any given point on the central axis from which photons scatter, there is a shadow region that singly scattered photons cannot reach (assuming opaque side walls).

**FIGURE 7:**
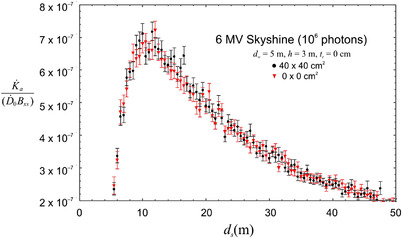
The scaled air kerma rate as a function of horizontal distance from the isocenter for a 6 MV broad beam (40 × 40 cm^2^) and a narrow beam (0 × 0 cm^2^) both based on 10^6^ photon histories. There is no discernible difference between the two cases showing that the skyshine is independent of the field size when the number of photons is the same. This shows that a strongly forward peaked beam (such as flattening filter free [FFF] beam) results in the same skyshine as a broad beam

The effect on the skyshine of an FFF beam has been evaluated by examining the extreme case in which all the photons are projected upward along the central axis of the beam (0 × 0 cm^2^, narrow beam). We have not accounted for any spectral changes that may be associated with FFF beams. A thick roof will tend to diffuse the narrow beam and act like a scattering foil. For this reason, a roof thickness of zero is expected to show the greatest difference. Figure 7 shows the skyshine AKR as a function of *d*_s_ for a 6 MV, 40 × 40 cm^2^ beam in comparison to a narrow beam (other parameters are [*d_w_*, *h*, *t_r_*] = [5 m, 3 m, 0 cm]). The number of photon histories (10^6^) is the same in both cases. This figure shows that there is no significant difference between the narrow beam and the broad beam. This result is likely due to the symmetry of the beam with respect to the central axis and the small opening angle of the beam. For the 40 × 40 cm^2^ beam, the *maximum* angle between an initial photon trajectory and the central axis is only about 13^o^. An FFF beam will of course have a higher dose rate than a beam with a flattening filter. It is suggested that the dose rate to use in Equation (1) for an FFF beam should be an average over the field.

For a “flat” field, the number of photons needed to maintain a fixed dose rate as the field size increases must be (to first order) proportional to the field area. As Figure 7 shows that the AKR is independent of field size for a fixed number of photon histories, we can conclude that the AKR is proportional to the field area for fixed dose rate as embodied in Equation (1). The AKR is not proportional to Ω ^1.3^ (Ω is the solid angle subtended by the beam) as predicted by the formula found in many references.^2^, ^4^, ^5^

The density of the air will have an effect on the skyshine. The density of dry air at sea level is 1.225 kg/m^3^ at 15°C, and this is the default standard density used here unless otherwise noted. At an altitude of 2000 m, the air density is 1.010 kg/m^3^. At higher elevation, where the density is lower, there will be less scattering, although at the same time, the radiation will experience less attenuation. In the limit of zero air density, there will be no skyshine. It has been shown that for monoenergetic photons singly scattered from the central axis (neglecting attenuation), the AKR is proportional to the air density.^8^ A comparison has been made for 10 MV beams with parameters (*d_w_*, *h*, *t_r_*) = (5 m, 3 m, 0 cm), 10^7^ photons, scoring plane at height 1.3 m above ground level, for field size 40 × 40 cm^2^ with an air density of 1.010 kg/m^3^ versus 1.225 kg/m^3^. The dimensionless AKR has been compared at *d_s_* = 11 m (*d*_max_ = 10.8 m). For the low density case, the AKR is (2.73 ± 0.04) × 10^−7^, and for the higher density, it is (3.26 ± 0.05) × 10^−7^. This shows that the AKR is highest at sea level and declines with elevation, scaling linearly with the air density (at least to first order). The values of *k* in Table 2 will overestimate the AKR at altitude.

## 4 DISCUSSION

If the roof is sufficiently thick, the skyshine will be below the background radiation level at *d*_max_. We assume an instantaneous background radiation level of 0.1 μSv/hr and nominal parameters (*d_w_*, *h*) = (5 m, 3 m), *F*_0_ = 1600 cm^2^, ${\dot{D}}_{0}$ = 600 cGy/min. Under these circumstances ${\left({\dot{K}}_{a}\right)}_{\max}$ < 0.1 μSv/hr when *B*_xs_ < 5.8 × 10^−4^ (>140 cm concrete) for 18 MV and *B*_xs_ < 3.3 × 10^−4^ (>110 cm of concrete) for 4 MV.

The weekly dose equivalent, in units of μSv/wk, for photon skyshine is given by:

(5) $$\begin{array}{c} H_{s}=1.5\times 10^{-2}k\left(W_{p}UT\right)\left(\frac{F_{0}}{400}\right)B_{xs}\frac{1}{d_{s}}\\ \left[2{\left(1+x^{2}\right)}^{3/2}-x\left(2x^{2}+3\right)\right], \end{array}$$

where *W_p_* is the weekly primary beam workload in Gy, *U* is the use factor for the roof, *T* is the occupancy for the location of interest outside the vault, *F*_0_ is the field area in units of cm^2^, *d_s_* is the horizontal distance from isocenter in meters and *x* = *h*/(*d_s_* −*d_w_*). McGinley and Martin have pointed out that the occupancy factor in Equation (5) may need to be as large as 1.0 in the event that there is a nearby single story building (presumably having no roof shielding), or if the property line is nearby.^4^ The “adjacent” building could actually be contiguous and could refer to an adjacent room within the same building. It is also presumed that *T* = 1 beyond the property line of a radiation therapy facility. The total radiation level will be *H_s_* plus whatever radiation penetrates the side wall.

Consider the case in which the roof is designed for occupancy with a weekly limit of 20 μSv/wk as recommended by NCRP151 for an uncontrolled area. The equivalent weekly dose at a height of 1 m above the roof top is given by ${H_{w,}}_{r}=B_{xs}\left(W_{p}UT_{r}\right)/{\left(h+2\right)}^{2}$, where *H_w,r_* and *W_p_* are expressed in the same units, and *h* is in units of meters. *W_p_* is the primary beam workload, and *T_r_* is the roof occupancy. If *H_w,r_* = 2.0 × 10^−5^ Sv/wk, then $\left(W_{p}UT_{r}\right)B_{xs}=2\times 10^{-5}{\left(h+2\right)}^{2}$. We can substitute this expression into Equation (5) assuming that the roof occupancy is *T_r_* = 1/20. For (*d_w_*, *h*) = (5 m, 3 m), and *F*_0_ = 1600 cm^2^, the maximum value of *H_s_* = 5.7 × 10^−5^ *k* in units of μSv/wk assuming that *T* = 1.0. This is independent of the individual values of the workload, use factor, and *B*_xs_, provided that the roof is shielded for occupancy. For 4 MV, *H_s_* = 0.009 μSv/wk and for 18 MV, *H_s_* = 0.005 μSv/wk. *If the roof is designed for occupancy, photon skyshine is utterly negligible*.

A “high radiation area” as defined by the US Nuclear Regulatory Commission is one in which the radiation level exceeds 1 mSv in a 1‐h time span. Such an area requires signage and restricted access control, which is certainly undesirable. Now consider the case in which the roof is not designed for occupancy but is designed to avoid designation as a high radiation area. The hourly dose equivalent is given by $H_{h,r}=W_{h}UB_{xs}/{\left(h+2\right)}^{2}$ where the hourly workload *W_h_* = *W_p_*/40. If *H_h,r_* = 10^−3^ Sv, then $W_{p}UB_{xs}=4\times 10^{-2}{\left(h+2\right)}^{2}$. This latter expression may be substituted back into Equation (5) (also using Equation (2)) to find the maximum skyshine. For (*d_w_*, *h*) = (5 m, 3 m), *F*_0_ = 1600 cm^2^, *H_s_* = 5.7 × 10^−3^ *kT* in units of μSv/wk. This is independent of the workload, use factor, and *B_xs_*, provided that the roof is shielded to avoid a high radiation area. If *T* = 1/20, the maximum value of *H_s_* is 0.04 μSv/wk (at *d*_max_ = 10.8 m). *If a roof is shielded adequately to avoid designation as a high radiation area then the photon skyshine will be negligible for low occupancy areas*.

The worst case scenario is the one in which there is no roof shielding. Let us first consider the case in which the area in question has an occupancy of *T* = 1.0 (an adjacent room) and is uncontrolled. If the beam energy is 4 MV, *W_p_* = 500 Gy, *F*_0_ = 1600 cm^2^ then (*H_s_*)_max_ = 110 μSv/wk for (*d_w_*, *h*) = (5 m, 3 m). This is about the highest skyshine AKR that one can reasonably imagine, and some roof shielding will be mandatory if the design goal is 20 μSv/wk. *Conclusion: if the area to be protected has T = 1 and is uncontrolled, then some roof shielding is always required*. On the other hand if *T* = 1/20 (NCRP151, outdoor areas with seating), then (*H_s_*)_max_ = 5.5 μSv/wk and the need for roof shielding will depend on the amount of radiation penetrating the side wall.

## 5 CONCLUSIONS

MC calculations of linac skyshine have been performed for a range of beam energies and vault dimensions. It has been shown that the skyshine AKR is conservatively predicted by an algebraic formula with a single energy dependent parameter. A quick and accurate method has been outlined to evaluate photon skyshine.

The following procedure is recommended for roof design and skyshine evaluation.

1. When it is feasible, design the roof to avoid designation as a “high radiation area” (1 mSv in any one hour). For nominal vault dimensions and workload, this will require *B*_xs_ ≲ 0.01. For 18 MV, this entails a minimum of 1.0 m of concrete. Under these circumstances photon skyshine is negligible for low occupancy areas.
2. When full roof shielding does not exist or is not feasible, use Equations (5) and (2) along with the values of *k* in Table 2 to estimate the maximum weekly skyshine outside the vault at a height of 1.3 m above level ground. The quantity *B*_xs_ must be calculated using the values of TVL_1_ and TVL_e_ in NCRP151. The roof composition and thickness are required for this calculation. Geometry definitions are shown in Figure 1. For an especially thin roof, (concrete thickness ≲30 cm) the lowest beam energy may lead to the largest AKR. The skyshine radiation must then be added to that penetrating the side wall.
3. Survey the skyshine. For the largest field size, find the distance from the outer wall (height 1.3 m) at which the skyshine AKR is highest. Equation (2) provides a prediction of this distance. If the roof is especially thin, the skyshine AKR may be highest for the lowest or intermediate beam energy. To measure the skyshine alone, the leakage radiation transmitted through the side wall must be subtracted. The leakage alone can be measured by closing the jaws as far as possible to eliminate the skyshine component. Background radiation levels should also be subtracted.

## CONFLICT OF INTEREST

The authors declare that there is no conflict of interest that could be perceived as prejudicing the impartiality of the research reported.

## Footnotes

* Equation (1) is derived from a similar, although more restricted integral formula, in the absence of any photon attenuation and assuming that the photon energy is much greater than 0.5 MeV.

† The Varian “Designers Desk Reference” (Vol. 16, No. 4, 2016)

‡ Elekta Oncology Products “Site Planning Guide” (2019)
